# Multi-gas quartz-enhanced photoacoustic sensor for environmental monitoring exploiting a Vernier effect-based quantum cascade laser

**DOI:** 10.1016/j.pacs.2022.100401

**Published:** 2022-09-05

**Authors:** Andrea Zifarelli, Raffaele De Palo, Pietro Patimisco, Marilena Giglio, Angelo Sampaolo, Stéphane Blaser, Jérémy Butet, Olivier Landry, Antoine Müller, Vincenzo Spagnolo

**Affiliations:** a PolySense Lab - Dipartimento Interateneo di Fisica, University and Politecnico of Bari, Via Amendola 173, Bari, Italy; b PolySense Innovations Srl, Via Amendola 173, Bari, Italy; c Alpes Lasers SA, Avenue des Pâquiers 1, 2072 St-Blaise, Switzerland

**Keywords:** QEPAS, Widely tunable QCL, Multi-gas sensing, Environmental monitoring

## Abstract

We report on a gas sensor based on quartz-enhanced photoacoustic spectroscopy (QEPAS) able to detect multiple gas species for environmental monitoring applications, by exploiting a Vernier effect-based quantum cascade laser as the excitation source. The device emission spectrum consists of ten separated emission clusters covering the range from 2100 up to 2250 cm^−1^. Four clusters were selected to detect the absorption features of carbon monoxide (CO), nitrous oxide (N_2_O), carbon dioxide (CO_2_), and water vapor (H_2_O), respectively. The sensor was calibrated with certified concentrations of CO, N_2_O and CO_2_ in a wet nitrogen matrix. The H_2_O absorption feature was used to monitor the water vapor within the gas line during the calibration. Minimum detection limits of 6 ppb, 7 ppb, and 70 ppm were achieved for CO, N_2_O and CO_2_, respectively, at 100 ms of integration time. As proof of concept, the QEPAS sensor was tested by continuously sampling indoor laboratory air and monitoring the analytes concentrations.

## 1 Introduction

The pressing demand for gas sensing solutions in the field of environmental monitoring relies on the multiple benefits that the protection of the environment reflects on human life and health [1], [2]. The monitoring of the air contaminants concentration leads to a better understanding of the emission processes and allows the mapping of the emission sources, for both outdoor and indoor environments [3], [4]. With this perspective, the analysis of air pollutants can be considered as a tool to assist decision making, and to develop strategies aimed at reverse negative environmental impacts [5]. Human activities lead to the emission of greenhouse gases and their precursors, which are the main responsible of global warming increase [6]. In this field, efficient monitoring activities require gas sensing technologies able to perform multi-gas detection [7]. In fact, the evaluation of multiple analytes allows the quantification of the mixing ratio variations as well as the analysis of the correlation among the gaseous emissions [8], [9]. Quantitative detection of gases has been traditionally performed with laboratory analytical equipment such as gas chromatographs and mass spectrometers [10], and with portable devices such as semiconductor gas sensors and electrochemical sensors [11]. Gas chromatography represents a solid benchmark for the analysis of gas samples, but its application is still limited in the case of real-time, in situ monitoring [12]. Conversely, gas sensors based on chemical reaction between the target analyte and the sensing element are suitable for in situ and real-time analysis, but their response is affected by long recovery time as well as poor selectivity and cross sensitivity [13]. Gas sensors based on optical detection techniques are a promising alternative for noninvasive measurements with high selectivity and sensitivity, being the perfect candidates for real-time, on-field operations [14]. Among the optical spectroscopic techniques, quartz-enhanced photoacoustic spectroscopy (QEPAS) has emerged as a reliable and robust technique for the detection of several gas species in trace concentrations [15], [16]. QEPAS technique represents an evolution of traditional PAS, exploiting a quartz tuning fork (QTF) to detect the weak sound waves produced by molecules absorbing modulated light. The prongs deflection induced by the pressure waves hitting the QTF is converted into an electric current by means of piezoelectric properties of quartz. Therefore, in QEPAS sensors the QTF acts as both detector and transducer, reducing the overall size of the detection module. In addition, a pair of millimetric resonator metallic tubes are typically used to amplify the acoustic waves intensity [17], and, together with the QTF, compose the QEPAS spectrophone. The key aspect of QEPAS is that the QTF response is independent of the light source wavelength employed to excite the gas target; thus the same detection module can operate with laser sources emitting in a wide range of wavelengths from UV to THz, making QEPAS an ideal technique for multi-gas detection [18], [19]. Therefore, the focus for multi-gas detection is completely shifted to the laser source. Multi-gas detection with standard DFB laser diodes is limited by the tunability range of the source, typically few cm^−1^ in the mid-IR for standard quantum cascade lasers (QCLs) and interband cascade lasers [20], [21], [22], [23]. Moreover, the selected absorption features can overlap, thus compromising the selectivity of the developed sensor and often requiring complex statistical tools to retrieve analytes concentrations [24]. Semiconductor laser sources with broader spectral emission, as arrays of QCLs or external cavity QCLs [25], [26], [27]. can be used to extend the emission spectral range, but the spectral selectivity is still restricted by the laser source resolution. An alternative approach is to use an array configuration, namely multiple laser sources emitting at different spectral ranges, each one targeting a single absorption feature, arranged in a single housing. Nevertheless, this approach complicates the sensor architecture requiring for additional sensing modules and complex spectrophone configuration [28], [29].

In this work, an innovative Vernier effect-based QCL was employed as the light source for a QEPAS sensor to detect multiple analytes with strong relevance for environmental monitoring, i.e., carbon monoxide (CO), nitrous oxide (N_2_O), carbon dioxide (CO_2_) and water vapor (H_2_O). The unique emission properties of this source allow the targeting of well-separated absorption features in a broad spectral range, providing high selectivity together with state-of-the-art sensitivity levels. The sensor calibration was performed on CO, N_2_O and CO_2_ in a wet nitrogen matrix. The H_2_O concentration in the gas line was fixed to prevent alterations in the QEPAS signal due to energy relaxation effects [30], [31]. Finally, the QEPAS sensor was tested by sampling indoor laboratory air and monitoring the analytes concentrations, as proof of concept.

## 2 Materials and methods

### 2.1 Laser source characterization

**Fig. 1:**
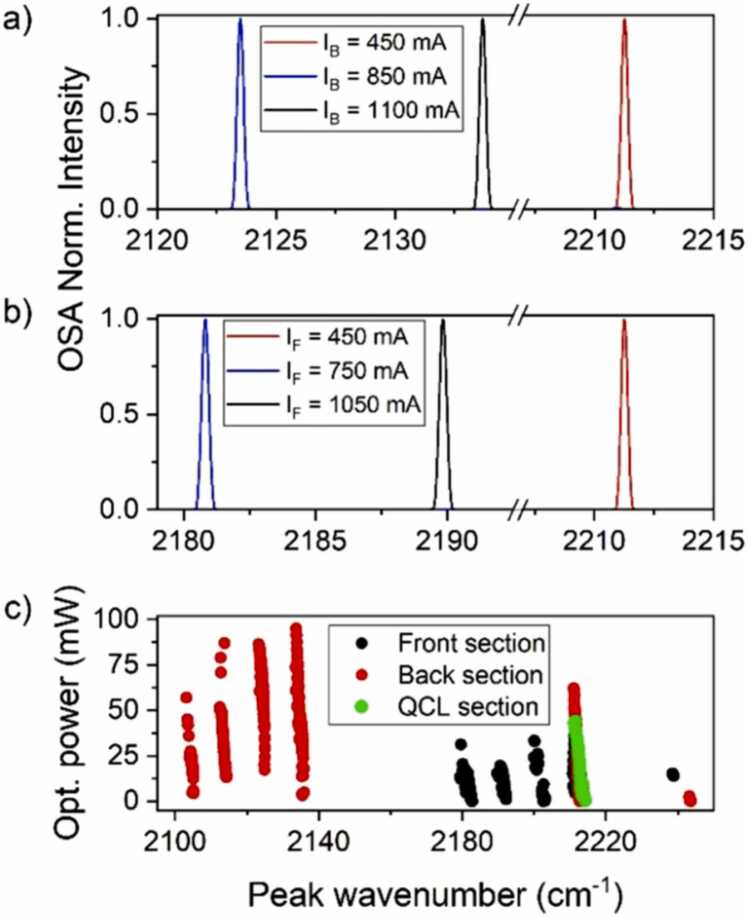
(a) Laser emission at I_L_ = 840 mA and different values of I_B_: 450 mA (red curve), 850 mA (blue curve), 1100 mA (black curve), while I_F_ = 0; (b) Laser emission at I_L_ = 840 mA and different values of I_F_: 450 mA (red curve), 750 mA (blue curve), 1050 mA (black curve), while I_B_ = 0; (c) Optical emission power as a function of the laser peak emission wavenumber for configurations employing the Front Section (black dots), the Back Section (red dots) and the QCL section (green dots).

The laser device employed as the light source for the QEPAS sensor was a custom Vernier effect-based QCL provided by Alpes Lasers. The Vernier effect was firstly developed by Pierre Vernier and is mainly known for its application in calipers. By employing two measurement scales with different periods, it is possible to improve the accuracy of a measurement by exploiting the overlap of the two scales. In a Vernier effect-based QCL, the grating on the top of the active region is designed following the same effect. Different configurations of integrated grating reflectors have been used to extend the emission range of laser sources by means of the Vernier effect [32], [33], [34]. However, the use of these designs in spectroscopic applications can be limited by the required sophisticated electronic driving [35]. The laser source employed in this work is characterized by a novel design employing two integrated heater resistors buried close to the active region. The semiconductor resistors are etched into the top cladding close to the QCL active region and act as local heaters to shift two reflectivity combs, representing the Vernier scales in the present case. By varying the current injected in the integrated heaters, the optical properties of the laser cavity are tuned. Indeed, the laser cavity is divided into two gratings, the so-called front and back gratings, the effective refractive of which can be tuned by injecting a current in the corresponding resistor, labeled as I_F_ for the front resistor and I_B_ for the back resistor, respectively. As a consequence, the emission wavelength can jump from one spectral region to another, over the spectral gain of the active region, as the alignment between the reflectivity combs evolves [36]. Once the amplitude of the current flowing in one of the heaters is fixed, a predictable emission can be obtained by varying the laser injection current in the active region, labeled I_L_. Several approaches have been used to design the front and back gratings, including optimization algorithms [36]. The device used in this work is composed of distributed Bragg reflectors (DBR), resulting in constant cluster jumps [37]. Therefore, the emission wavelength of the QCL can be selected using a specific combination of I_F_, I_B_ and I_L_ and tuned to address specific absorption lines. As a first step, the spectral emission of the laser device was characterized employing an optical spectrum analyzer (Thorlabs OSA207C) with a spectral resolution of 0.25 cm^−1^. The laser temperature was set to 0 °C, employing a water cooler (ThermoCube 200 W) as a thermal bath and driving the Peltier-effect cooler integrated in the laser packaging with the Arroyo 5300 Series thermo-electrical cooler (TEC). This temperature was set throughout all the measurements. The two heaters were alternatively switched on, thus providing two different electrical configurations: (I_L_, I_F_, I_B_ = 0) or (I_L_, I_F_ = 0, I_B_). These configurations were explored by varying both the heater current (I_F_ or I_B_) and the laser injection current (I_L_), mapping the emission range of the device and acquiring the corresponding output power. The laser was controlled using three laser current drivers, one for each section. The QCL section was driven using an ILX Lightwave LDX-3232, while two Arroyo 4300 Series current drivers were used to inject current into the Front and Back sections. With I_L_ varying in its dynamic range from 590 mA to 850 mA, I_F_ was varied from 450 mA to 1100 mA while I_B_ = 0; conversely, I_B_ was varied from 450 mA to 1100 mA while I_F_ = 0. The device was also operated as a traditional QCL with the configuration (I_L_, I_F_ = 0, I_B_ = 0). As representative, the laser emissions corresponding to six electrical configurations are shown in Fig. 1a–b. These spectra were acquired by setting I_L_ = 840 mA, and I_F_ (Fig. 1a) or I_B_ (Fig. 1b) at three different values.

The whole laser emission range is reported in Fig. 1c. For each collected spectrum, the center wavenumber was extracted and is plotted together with the corresponding optical power value. All measurements reported in Fig. 1a–c are referred to spectra with single-mode emission. With the configuration (I_L_, I_F_, I_B_ = 0), five well-separated spectral ranges can be clearly identified (black dots in Fig. 1c). These five regions have the central emission wavenumber at 2180 cm^−1^, 2190 cm^−1^, 2205 cm^−1^, 2212 cm^−1^ and 2239 cm^−1^, respectively. With the configuration (I_L_, I_F_ = 0, I_B_), other six well-separated spectral ranges can be covered by varying I_B_ and I_L_ (red dots in Fig. 1c). These ranges are characterized by a central emission wavenumber at 2105 cm^−1^, 2113 cm^−1^, 2125 cm^−1^, 2135 cm^−1^, 2212 cm^−1^ and 2243 cm^−1^, respectively. Operating the device as a traditional QCL (I_L_, I_F_ = 0, I_B_ = 0), a spectral emission range characterized by a central emission wavenumber at 2212 cm^−1^ was observed (green dots in Fig. 1c). Therefore, all the three employed configurations are spectrally overlapped in this range, resulting in ten well-separated spectral regions covered by the Vernier effect-based QCL.

### 2.2 Experimental setup

**Fig. 2:**
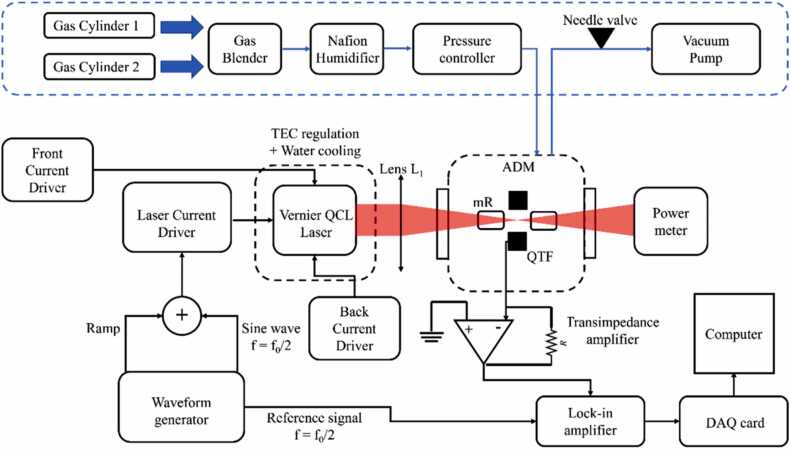
Schematic of the employed experimental setup. QTF, quartz tuning fork; mR, resonator tube; ADM, acoustic detection module.

A schematic of the QEPAS sensor developed using the Vernier effect-based QCL is shown in Fig. 2. The collimated laser beam exiting the device was focused through the acoustic detection module (ADM) by means of a CaF_2_ plano-convex lens with focal length of 50 mm (Thorlabs LA5763-E). The size of the beam spots obtained when changing the device electrical configuration was preserved, and no significant differences were observed in the lens focal plane. The employed ADM was a stainless-steel vacuum-tight chamber equipped with two wedged ZnSe windows with an anti-reflection coating in the 2–13 µm range (Thorlabs WG 80530-E). Inside the ADM, the QEPAS spectrophone consisted of a T-shaped QTF acoustically coupled of a pair of resonator tubes. All geometrical parameters and the assembly of the spectrophone are reported in Ref. [17]. The spectrophone had a fundamental resonance frequency at f_0_ = 12,458.1 Hz with a quality factor Q = 11,900 at atmospheric pressure (P = 760 Torr). The light exiting from the ADM was then collected by a power meter for alignment purposes.

QEPAS measurements were performed in *2f*-wavelength modulation spectroscopy (WMS), namely the laser emission was modulated by applying a sinusoidal waveform to the injection current I_L_ at a frequency f = f_0_/2. This modulation was generated by a waveform generator (Tektronix AFG3021C), providing also a low-frequency triangular ramp used to scan across the targeted absorption line. The piezoelectric current generated by the QTF was converted into a voltage signal using a transimpedance preamplifier (gain factor = 30, R_fb_ = 10 MΩ) [38], and then was sent to a lock-in amplifier (EG&G 7265) to be demodulated at f = f_0_. The lock-in time constant was set to 100 ms for all measurements reported in this work. The lock-in amplifier analog signal was digitalized by a National Instruments DAQ card (USB 6008) connected to a personal computer. The collected QEPAS signal was then recorded using a LabVIEW-based software. The gas line was composed of certified concentrations gas cylinders, a gas mixer, a humidifier, a pressure controller, a system of needle valves and a vacuum pump. The QEPAS sensor calibrations were performed using the following certified concentrations: 10 ppm CO in N_2_, 10 ppm N_2_O in N_2_ and 7000 ppm CO_2_ in N_2_. One at a time, they were connected to the inlet of the gas mixer, together with a cylinder containing pure nitrogen for successive dilutions. The pressure of the gas mixture flowing inside the ADM was regulated using a pressure controller (MKS Type 649), while the flow rate was set by the gas mixer (MCQ Instruments, Gas Blender 103). A Nafion humidifier (PermSelect PDMSXA 1 cm^2^) was placed after the gas mixer to humidify the gas samples, fixing the water vapor concentration for all measurements. The humidity level within the gas line was verified using a capacitive hygrometer (not shown in Fig. 2). The sensor was operated at room temperature T_room_ = 25 °C, during all the measurement sessions.

## 3 Results and discussion

### 3.1 QEPAS sensor characterization

**Fig. 3:**
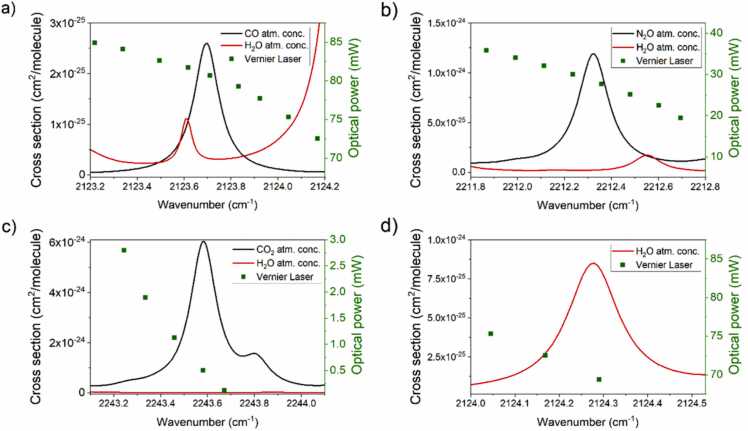
Absorption cross section of target analytes at atmospheric concentration simulated using the HITRAN database (solid curve) and laser optical power within the simulation spectral ranges (green squares). a) 150 ppb of CO (black curve) and 1.19 % of H_2_O (red curve) in N_2_; b) 310 ppb of N_2_O (black curve) and 1.19 % of H_2_O (red curve) in N_2_; c) 330 ppm of CO_2_ (black curve) and 1.19 % of H_2_O (red curve) in N_2_; and d) 1.19 % of H_2_O (red curve) in N_2_. All the spectra were simulated at atmospheric pressure.

Four gases relevant for environmental monitoring exhibit absorption features within the laser full emission range: carbon monoxide (CO), nitrous oxide (N_2_O), carbon dioxide (CO_2_) and water vapor (H_2_O). The absorption cross sections of the analytes within the laser emission range were simulated using the HITRAN database [39] and are plotted in Fig. 3 (solid lines) together with the corresponding laser optical power (green squares). The spectra were simulated at atmospheric pressure for mixtures in nitrogen containing the typical analytes concentrations in air, taking as reference the values of standard air sample reported on HITRAN database (H_2_O: 1.19 %; CO_2_: 330 ppm; N_2_O: 310 ppb; CO: 150 ppb). The selected CO absorption feature peaked at 2123.69 cm^−1^ with a close H_2_O absorption peak at 2123.6 cm^−1^ is shown in Fig. 3a. In this spectral region, the laser optical power varies from 69 to 80 mW, corresponding to the device configuration: I_F_ = 0, I_B_ = 800 mA, I_L_ = 800–860 mA. The selected N_2_O absorption feature is peaked at 2212.35 cm^−1^ with a close H_2_O absorption peak at 2212.57 cm^−1^, as shown in Fig. 3b. In this spectral region, the laser optical power varies from 20 to 35 mW, corresponding to the device configuration: I_F_ = 0, I_B_ = 0, I_L_ = 730–790 mA. The selected CO_2_ absorption feature is peaked at 2243.59 cm^−1^ isolated from any interferent present in atmosphere, as shown in Fig. 3c. In this spectral region, the laser emission power ranged from 0.1 to 3.5 mW, corresponding to the device configuration: I_F_ = 0, I_B_ = 450, I_L_ = 620–660 mA. The selected H_2_O absorption feature peaked at 2124.29 cm^−1^ is shown in Fig. 3d. In this spectral region, the laser emission power ranges from 70 mW to 75 mW, corresponding to the device configuration: I_F_ = 0, I_B_ = 800, I_L_ = 760–800 mA. A preliminary investigation was performed to determine the best operating pressure for the QEPAS sensor, which was found to be P = 450 Torr. This value was selected as it provided the highest QEPAS signal for CO, which is the gas species with the lowest atmospheric concentration among the investigated gas species. The gas flow rate was fixed at 60 sccm to keep a constant 2 % water concentration within the gas line. The need for a stable water concentration comes from the well-known dependence of the photoacoustic signal on the presence of energy relaxation promoter, such as H_2_O. This effect was already observed for the target gases in the selected spectral regions [40], [41], [42]. Therefore, a fixed water vapor concentration was required to perform an efficient calibration of the QEPAS sensor. During the measurements, the water vapor concentration was monitored using the QEPAS signal measured at the absorption peak shown in Fig. 3d.

**Fig. 4:**
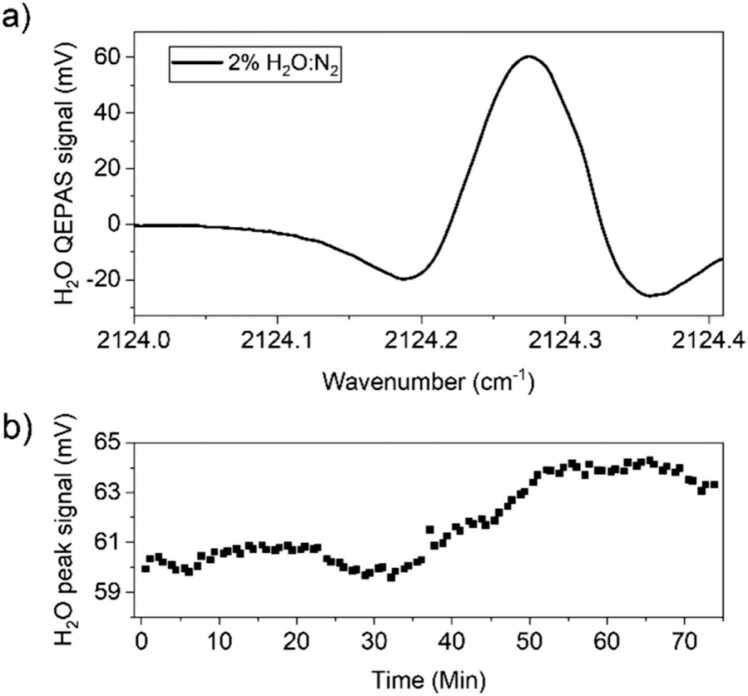
(a) *2f*-QEPAS spectral scan of the H_2_O absorption feature at 2 % concentration in N_2_; (b) peak values from a 75-min-long acquisition of QEPAS signal for 2 % H_2_O in N_2_.

The *2f*-QEPAS spectral scan corresponding to a concentration of 2 % H_2_O in N_2_ acquired scanning across the water absorption peak (see Fig. 3d) is shown in Fig. 4a. This humidity level was selected to remove the effects of environmental water fluctuations as it was higher than the H_2_O concentration measured in our laboratories. The QEPAS spectrum reported in Fig. 4a matches the expected sensor response, since it retraces the 2^nd^ derivative of the Lorentzian absorption feature shown in Fig. 3d. The right-hand side negative lobe is only partially reconstructed due to narrow spectral range covered by the device configuration, as shown in Fig. 3d. However, the positive lobe is completely defined, allowing the extraction of the peak value which is, in turn, related to the actual water concentration within the ADM.

**Fig. 5:**
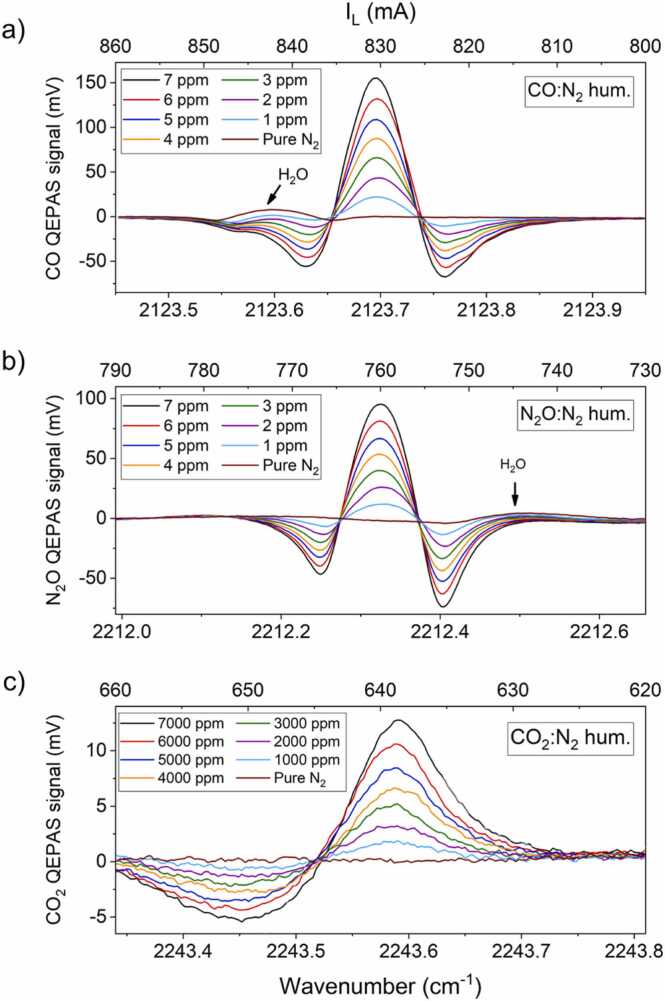
(a) *2f*-QEPAS spectral scans of the CO absorption feature at eight CO concentrations; (b) *2f*-QEPAS spectral scans of the N_2_O absorption features at eight N_2_O concentrations; (c) *2f*-QEPAS spectral scans of the CO_2_ absorption peaks at eight CO_2_ concentrations.

No calibration of the H_2_O QEPAS signal has been performed since we decided to keep the water concentration at 2 % via the humidifier and no significant variations of the values were expected. To verify the long-term stability of the humidifier, subsequent spectral scans were performed over a 75-min-long acquisition; then, the QEPAS peak signals were extracted and plotted as a function of time in Fig. 4b. The collected data show relative fluctuations within an interval of ± 6 % of the mean value of the H_2_O QEPAS signal over more than one hour. The observed fluctuations are related to the operating principle of the employed humidifier, consisting in hollow fibers releasing water vapor molecules in the gas flow passing through. Slight variations in the thermo- and fluid-dynamics parameters in the gas line can lead to small fluctuations in the H_2_O released in the gas flow. Moreover, the collected trend shows 30-min-long acquisitions characterized by a relative standard deviation of ∼ 1 % indicating a good humidification stability. Such low variations of water concentration do not significantly affect the QEPAS signal of the target gas species [40], [41]. Then, the QEPAS sensor was calibrated for CO, N_2_O and CO_2_ detection. The gas blender was used to generate several dilutions in humidified N_2_ of each gas target independently, starting from the certified concentrations available in the gas cylinders. The *2f*-QEPAS spectral scans collected at different gas target concentrations are shown in Fig. 5a–c for the three analytes.

**Fig. 6:**
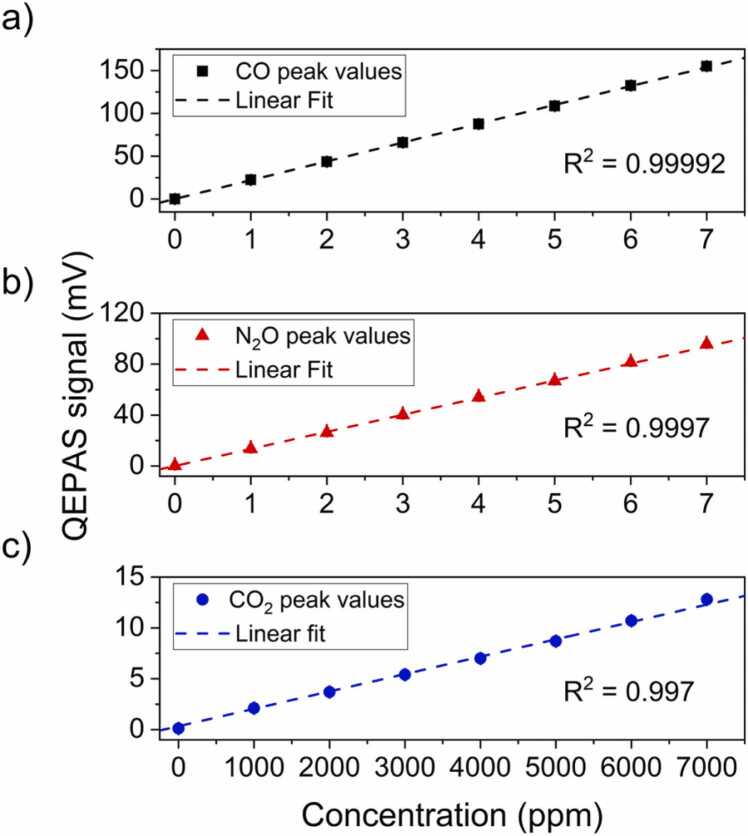
(a) QEPAS peak signals as a function of the CO concentration (black squares) and the corresponding best linear fit (black dashed line); (b) QEPAS peak signals as a function of the N_2_O concentration (red triangles) and the corresponding best linear fit (red dashed line); (c) QEPAS peak signals as a function of the CO_2_ concentration (blue dots) and the corresponding best linear fit (blue dashed line).

The *2f*-QEPAS spectra collected targeting CO at concentrations ranging from 0 to 7 ppm in humidified N_2_ are shown in Fig. 5a. The spectra were acquired tuning the I_L_ current in the 800–860 mA range while fixing the I_B_ current to 800 mA, as previously reported. The shape of the collected scans retraces the 2nd derivative of the Lorentzian lineshape with a small alteration on negative lobes due to the residual amplitude modulation contribution. The contribution to the overall spectra given by the H_2_O absorption feature peaked at 2123.6 cm^−1^ (see Fig. 4a) is clearly visible, causing a slight distortion on the left hand-side negative lobe of the *2f*-Lorentzian lineshape, without affecting the CO-QEPAS peak signal. In Fig. 5b the *2f-*QEPAS spectra for N_2_O concentrations ranging from 0 to 7 ppm in humidified N_2_ are shown. The spectra were acquired tuning the I_L_ current in the 730–790 mA range, while fixing both the I_B_ current and the current I_F_ to 0 mA. Neither the N_2_O absorption profile nor the N_2_O-QEPAS peak signal are affected by the H_2_O absorption peak at 2212.59 cm^−1^. The asymmetry of the negative lobes is due to the residual amplitude modulation contribution. In Fig. 5c the *2f-*QEPAS spectra of CO_2_ for concentrations ranging from 0 to 7000 ppm in humidified N_2_ are shown. The spectra were acquired tuning the I_L_ current in the 620–660 mA range, while fixing the I_B_ current to 450 mA. The collected QEPAS spectra partially retrace the 2nd derivative of a Lorentzian lineshape. In fact, the right-hand side negative lobe could not be reconstructed since the lasing threshold current for the employed device configuration was I_L_ *=* 620 mA (corresponding to 2243.7 cm^−1^, as shown in Fig. 5c), thus low optical powers were provided by the injection currents nearby. The peak values of each QEPAS spectrum reported in Fig. 5 were extracted and reported in Fig. 6 as a function of the corresponding gas target concentrations. For each dataset, the dashed line represents the best linear fit of the experimental data.

For each gas target a 1σ-fluctuation of 2 % of the peak value was estimated for each target concentration. In Table 1, the results of the linear fits, namely the slope (the sensitivity) and the intercept, with the noise level, the estimated minimum detection limit (MDL) and the normalized noise equivalent absorption (NNEA).

**Table 1:** Sensor calibration results for the target analytes.

	Sensitivity (mV / ppm)	Intercept (mV)	Noise (mV)	MDL	NNEA (W cm^−1^^/^ √Hz)
**CO**	21.96 ± 0.06	0.13 ± 0.05	0.13	6 ppb	6.3 · 10^−9^
**N**_**2**_**O**	13.45 ± 0.06	0.08 ± 0.08	0.10	7 ppb	5.4 · 10^−9^
**CO**_**2**_	1.73 · 10^−3^ ± 0.04 · 10^−3^	0.30 ± 0.11	0.12	70 ppm	5.0 · 10^−9^

For each of the three analytes, the noise level was estimated as the 1σ-fluctuation of the QEPAS signal acquired while pure N_2_ was flowing in the ADM and the sine-modulated injection current was fixed at absorption peak. The minimum detection limits are the concentrations at which the signal-to-noise-ratio is equal to 1, for each gas species.

### 3.2 Indoor air quality monitoring

**Fig. 7:**
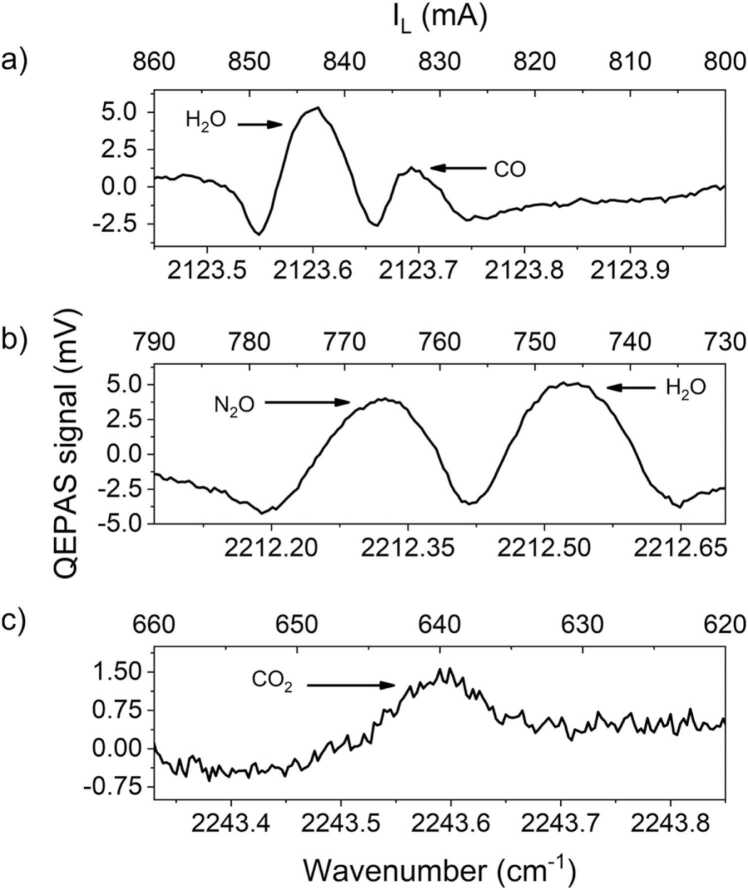
(a) *2f*-QEPAS spectral scan of CO absorption feature at atmospheric concentration in indoor environment; (b) *2f*-QEPAS spectral scan of N_2_O absorption feature at atmospheric concentration in indoor environment; (c) *2f*-QEPAS spectral scan of CO_2_ absorption feature at atmospheric concentration in indoor environment. *2f*-QEPAS spectral scan of H_2_O at a concentration of 2 % can be observed in Figs. (a) and (b).

As proof of concept, the developed QEPAS sensor was tested by sampling ambient air in a closed environment, i.e., the laboratory air. For each gas, the operating parameters were the same as the ones employed for calibration (P = 450 Torr, flow rate = 60 sccm), and the air samples humidity level was kept fixed around 2 % by fluxing the gas samples through the Nafion humidifier. The *2f*-QEPAS spectral scans acquired targeting the selected gas species in the laboratory air are shown in Fig. 7.

**Fig. 8:**
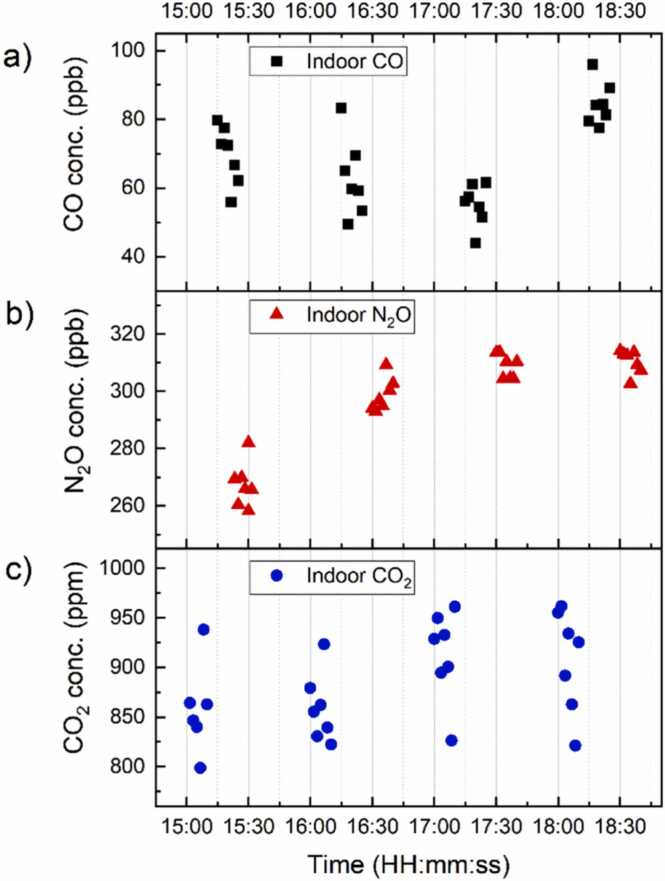
Target gases concentrations estimated during the long-term monitoring of indoor laboratory air. (a) Estimated CO concentration (black squares); (b) Estimated N_2_O concentration (red triangles); (c) Estimated CO_2_ concentration (blue dots).

The CO and N_2_O QEPAS spectral scans exhibit an adjacent water vapor absorption contribution, as expected from simulations reported in Fig. 3a and b, respectively. Nevertheless, the CO and N_2_O contributions are well resolved and separated, therefore the peak values used to retrieve the analytes concentration are still reliable. The CO_2_ QEPAS spectrum in Fig. 7c shows an analogous shape compared to those reported in Fig. 5c, with a negative lobe difficult to be identified. Long-term measurements of the three target gases were performed in two consecutive afternoons in late spring (26th and 27th of May 2021). Each analyte was continuously targeted for ten minutes before switching to the next one. This 40-min-long measurement was repeated four times, both the afternoons, for a total operating time of ∼ 4 h (from 15:00 to 19:00). The analytes were acquired in the following order: CO_2_, CO, N_2_O and H_2_O. From the acquired scans, the QEPAS peak values were extracted and converted into gas concentration employing the calibration curved shown in Fig. 6a–c. The target gases concentrations collected during day #1 are reported as a function of the acquisition time in Fig. 8.

The CO concentration trend (Fig. 8a) showed an increase during the whole measurement session, with a minimum of 40 ppb around 17:15 and a maximum of 98 ppb around 18:15. The N_2_O concentration trend (Fig. 8b) pointed out a rapid increase between the first and the second round of measurements. Then, the concentration stabilized around 315 ppb with a slight decrease until the end of the measurement session. The CO_2_ concentration trend (Fig. 8c) showed a variation of ∼ 160 ppm during the whole measurement session, with a minimum of 800 ppm around 15:05 and a maximum of 960 ppm around 18:00. The measurements reported in Fig. 8 were repeated the next day under the same experimental conditions, and the average concentrations of CO, N_2_O and CO_2_ are reported in Table 2 together with the corresponding 1σ standard deviation.

**Table 2:** Estimated average analytes concentrations in laboratory air samples.

	Avg. concentration day 1	Avg. concentration day 2
**CO**	64 ± 14 ppb	65 ± 21 ppb
**N**_**2**_**O**	296 ± 18 ppb	294 ± 22 ppb
**CO**_**2**_	891 ± 57 ppm	855 ± 68 ppm

The mean concentration values estimated in day #1 and day #2 show a good repeatability, as expected since the environmental conditions were similar during the two days of measurements. The average values of CO concentration estimated in laboratory air are comparable to those reported by the apulian regional agency for environmental protection (ARPA) [43]. The typical atmospheric CO concentration is taken as a reference since no CO sources were present in the laboratory during measurements [44]. In late spring days, the concentration may reach up to 200 ppb near to the monitoring station placed close to traffic hotspots. Considering the position of our laboratory, far from traffic jam and below the ground level, a lower concentration was expected. The average values of N_2_O concentration measured in laboratory are consistent with the typical atmospheric concentration of ∼ 330 ppb [6] but slightly lower. The average values of CO_2_ concentration are almost double compared to the typical outdoor CO_2_ concentration ∼ 400 ppm [45], but the measured values are consistent with the typical indoor CO_2_ concentration [46]. In fact, in closed environments, the CO_2_ levels strongly depend on the carbon dioxide emitted in human breath (∼ 4–5 % of total exhalation) [47]. Therefore, for indoor environments, the CO_2_ concentration can rapidly arise over the recommended daily limit of 1500 ppm depending on the number of people in the room and on its ventilation conditions [48]. The average estimated concentrations of CO_2_ are well below recommended daily limit and are compatible with a quite-well ventilated room with less than two occupants, which were the operating conditions inside the laboratory due to COVID-19 restrictions.

## 4 Conclusions

In this work, a multi-gas QEPAS sensor employing a QCL source based on Vernier effect for environmental monitoring applications was demonstrated. The device spectral emission consists of several clusters spanning from 2100 up to 2250 cm^−1^; four clusters were selected to reconstruct the absorption features of four different analytes: CO, N_2_O, CO_2_ and H_2_O. The developed sensor was calibrated in a wet N_2_ matrix with fixed H_2_O concentration. Several dilutions of CO, N_2_O and CO_2_ were performed, and a linear response of the sensor was observed for each analyte. Minimum detection limits of 6 ppb, 7 ppb, and 70 ppm were estimated for CO, N_2_O, and CO_2_, respectively, at 100 ms integration time. The achieved detection limits were all below the natural abundance of the gas species in atmosphere. Therefore, as proof of concept, the sensor was tested by sampling indoor laboratory air and monitoring the analytes concentrations in real-time. The measurements were repeated in two consecutive days, returning average estimated concentrations compatible with the expected atmospheric concentrations in an indoor environment.

## Declaration of Competing Interest

The authors declare that they have no known competing financial interests or personal relationships that could have appeared to influence the work reported in this paper.

## Data Availability

Data will be made available on request.
